# Hearing the voices of older adult patients: processes and findings to inform health services research

**DOI:** 10.1186/s40900-019-0143-5

**Published:** 2019-02-21

**Authors:** Sally Fowler Davis, Anne Silvester, Deborah Barnett, Lisa Farndon, Mubarak Ismail

**Affiliations:** 1 Sheffield Hallam University and Sheffield Teaching Hospitals NHS Foundation Trust, Montgomery House, 32 Collegiate Crescent, Sheffield, S10 2BR UK; 2 Sheffield Teaching Hospitals NHS Foundation Trust, Sheffield, UK; 3 Sheffield Hallam University, Sheffield, UK

**Keywords:** Frail older adults, Patient and public involvement and engagement (PPIE), Discharge planning, Patient experience

## Abstract

**Plain English summary:**

Whilst Patient and Public Involvement and Engagement (PPIE) are widely regarded as critical to developing clinical research, there is a perception that older adults may not be able to contribute and there is less emphasis on gaining a wide range of opinions before developing research questions or projects; for example an organisational change. This PPIE initiative used three PPIE processes including existing panels and wider networking to access older adults in the community who had used the hospital services and been discharged. Older adults expressed a range of views about their experience of discharge planning and this provided an important perspective on patients’ research priorities associated with their personal independence. Efforts were taken to ensure representative views across a cross section of the population. As a result of this initial PPIE, a permanent, co-ordinated ‘elders’ panel has been established to ensure a representation of older adult views for research, service development and evaluation. This panel has permanent, fully supported members who provide reflection and feedback on any projects and programmes relating to older people’s services in the City.

**Abstract:**

**Background**

Clinical academic research and service improvement is planned using Patient and Public Involvement and Engagement (PPIE) but older PPIE participants are consulted less often due to the perception that they are vulnerable or hard to engage.

**Objectives**

To consult frail older adults about a recently adopted service, discharge to assess (D2A), and to prioritise services improvements and research topics associated with the design and delivery of discharge from hospital. To use successive PPIE processes to enable a permanent PPIE panel to be established.

**Participants**

Following guidance from an established hospital PPI panel 27 older adult participants were recruited. Participants from Black, Asian and Minority Ethnic (BAME) communities, affluent and non-affluent areas and varied social circumstances were included.

**Methods**

Focus groups and individual interviews were conducted in participants own homes or nearby social venues.

**Results**

Priorities for discharge included remaining independent despite often feeling lonely at home; to remain in hospital if needed; and for services to ensure effective communication with families. The main research priority identified was facilitating independence, whilst establishing a permanent PPIE panel involving older adults was viewed favourably.

**Conclusions**

Taking a structured approach to PPIE enabled varied older peoples’ voices to express their priorities and concerns into early discharge from hospital, as well as enabling the development of health services research into hospital discharge planning and management. Older people as participants identified research priorities after reflecting on their experiences. Listening and reflection enabled researchers to develop a new “Community PPIE Elders Panel” to create an enduring PPIE infrastructure for frail older housebound people to engage in research design, development and dissemination.

## Introduction and background

This paper reports on a community-based initiative to enable the voice of frail older people to inform research and service development within the National Health Service (NHS) in England. A frail older adult in this paper refers to a person over 75, with some mobility issues and a wide range of health conditions and care needs. Patient and Public Involvement and Engagement (PPIE) in health and social care can be for the purpose of planning research and to understand the priorities of service users and here, both approaches were used [1]. The goal was to understand patient experience to generate research that improved services and produced better patient outcomes [2]. Involvement and engagement of a wide variety of older people was sought in a large provincial city in England, across a range of community settings. Group and individual meetings were arranged via key contacts and links with representative community leaders to access affluent and non-affluent areas as well as people from Black, Asian Minority Ethnic (BAME) Communities.

Consultation with a growing older adult population is an important agenda for the National Health Service (NHS) in terms of research and for the purpose of service development. A series of PPIE activity enabled successful recruitment in terms of numbers and the diversity of those consulted and a permanent PPIE group to consult on future research design and processes.

The acknowledged benefits of consulting older people include facilitation of health technologies, illumination of areas of practice leading to improvements in care, enhancement of both the quality of research and its potential for helping address more intractable health problems, and improving the chances of more relevant research being conducted [3–8]. Frail older house-bound people can unwittingly be excluded from consultation because, for practical reasons, they are less accessible. A further possible constraint to community participation is those being most sought and required are often “least likely to be in a position to donate their time and energy” [8]. The literature also suggests that traditional PPIE panels are often conducted via focus groups [5, 9], but the population of frail older people (particularly aged 80 and over) may have multiple health, mobility or vision difficulties and are unlikely to respond well to written questionnaires, telephone consultations or one off group meetings. Consensus on the preferred method of consultation with older people is for individual face-to-face home interviews [10]. It is suggested that using gatekeepers such as local clinicians and/or community leaders to access more commonly excluded groups, such as older adults, can be a helpful means of recruitment and developing trust [5, 11].

Greater involvement of users, carers and service providers in the design, delivery and evaluation of research associated with services for older people is now widely promoted [7] with a corresponding need to enable robust consultation and communication about critical issues in health and care planning. This paper describes how frail older people were engaged in discussions about discharge from hospital, identifying their issues and concerns and leading to a detailed understanding that could be used to focus research and service change. This initiative resulted in an ongoing Community Elders PPIE Panel.

### Health services for older adults

The frail older population comprise an increasing proportion of the United Kingdom (UK) population with 17.7% of adults aged over 65, a figure that is set to rise [12]. Within Europe those aged 80 and over will be the fastest growing age group, increasing from 5 to 12% between 2010 and 2060 [13]. Previous research has indicated that those who are frail and older are more at risk from adverse health outcomes, making it important that future research is conducted to improve and identify outcomes that are both health improvements and service improvements [5, 14].

An increasing problem in regard to frail older patients is delayed discharge from hospital, a trend that is rising. This is illustrated by the National Audit Office who calculated a 31% rise in delayed transfers of care between 2013 and 2015, with an estimated £820 million cost to the NHS of older patients in hospital beds who are no longer in need of acute treatment [15]. Longer hospital stays can lead to worse health outcomes, particularly relevant to the older adults, who also have the additional likelihood of increased long term care needs [15]. Acknowledged adverse outcomes from delayed hospital discharge include medical complications, infections, loss of mobility, reduced capability of carrying out daily living tasks, or even death [15]. In addition they can have a negative effect on patients’ health after discharge, increasing their chances of readmission to hospital [16]. Frail older patients also express their desire to be discharged in a timely manner, although with the caveat that they are physically well enough and have sufficient support at home to be able to cope [17].

Health service research and the changing models of care promoted by NHS England (NHS England 2015) have produced recommendations that discharge planning should begin as soon as possible after older adult patients are admitted into an acute hospital bed. Furthermore, a “discharge to assess (D2A)” model should be implemented, where older patients are assessed for care and equipment needs at home, rather than waiting in hospital for rehabilitation and social care assessment. Thus hospitalised older patients receive less therapy in hospital [18] and return home to receive community care [19]including nursing, therapy and home support. It is suggested this is the default pathway, with alternative pathways for those who cannot return home immediately [20].

### Funding for patient and public involvement and engagement

In July 2014 a Patient and Public Involvement and engagement (PPIE) project was funded with a small grant awarded by the Yorkshire and Humberside Research Design Service, National Institute for Health Research (NIHR). This award enabled access to an established PPIE group to advise on accessing a range of older patients’ voices regarding D2A, alongside supporting the preparation of a clinical fellowship application (see NIHR Clinical academic career fellowships) [21, 22]. This project was the first initiative to seek patient and public views into discharge management in the city wide D2A service and incorporate these into future improvements.

### Ethics and governance

As a PPIE initiative, this project was deemed to be exempt from ethics by the clinical research office in the sponsoring NHS Trust. The work was registered as a service review and carried out by clinical academics working within the Trust, seeking the views on elder research and furthering the development of a sustainable PPIE infrastructure. The funders who are Regional Design Service of the NIHR in Yorkshire and Humber (RDS Y&H) recognised PPIE as service review activity.

## Methods

### Design

An established NHS PPIE group, consisted of patient representatives from the local area and a co-ordinator post was hosted in a research team. The PPIE members were experienced in advising researchers about the methodology and burden of research activities, so they were approached. The PPIE panel were advised of the primary problem; how to access frail older participants who had experience of ward services and were willing to reflect on their experiences of discharge from hospital. The PPIE panel recommended that due to the limited funding of the £500 NIHR grant, the researchers should approach existing groups, social clubs and networks that attracted regular participation from frail older adults. Based on this advice, existing and known groups across a range of demographics were targeted to seek diverse members of the older adult population, including those whose opinions are rarely sought. The inclusion of different groups from BAME backgrounds and from deprived communities and those who may be less sociable or more isolated became part of the formal plan. The goal was to identify eight frail older participants who by virtue of a recent admission or their family carer role could provide a range of opinion into medical and care needs on discharge.. Beyond this discharge project, the initial PPIE activity was to be extended to involve older adults as stakeholders within the city’s research community, helping to direct and prioritise research more widely.

### Recruitment

Representatives of community groups and specific contacts were asked to identify any older members who may be interested in taking part in a PPIE focus group or interview in order to talk about their experiences of hospital discharge, especially D2A, and priorities around discharge. Using gatekeepers such as local clinicians or community leaders to access more commonly excluded groups was seen as a helpful means of recruitment and developing trust [5, 18]. Interested parties were provided with an information sheet about the project, which included the topic questions which were to be used for the discussions (Appendix). Volunteers who agreed to take part were given an appointment to talk individually or be part of a focus group in a place of their choice. Most preferred to be visited at a community venue but not a hospital site with just two preferring to be in their own home. Individuals and groups were invited to participate with support for travel, refreshments and any untoward costs associated with room hire. This enabled small organisations to facilitate by inviting members and potential participants and offering hospitality on behalf of the PPIE initiative.

#### Participants and attention to diversity

Contacts and groups approached included a retirement village, a BAME community group, a lunch club in a non-affluent area and an over 50s club in an affluent area. Nine older female participants were recruited from the retirement village and agreed to take part in a focus group in one of their community rooms. A researcher colleague with a prior relationship with the Pakistani Muslim Centre (PMC) approached the current manager who helped to identify thirteen men of Pakistani, Yemeni and Somali origin willing to take part. Assistance was provided by PMC leaders to enable consideration to be given to language and methods of asking for informed consent. The group took place within the PMC to ensure familiarity and engender trust. A chair of an over 50’s club in an affluent area was approached and facilitated a discussion within a local church hall. Although more than 30 people were present, only three women actively participated. Finally two members from an established lunch club in a non-affluent area volunteered to home-based interviews.

#### Participant focus groups and interviews

To ensure common areas of questioning and to explore specific issues a list of topic questions guided the researchers who facilitated the groups or individual discussions [14] (Appendix). It enabled participants to share their experience and reflection on hospital care and need for supported discharge, and focus possible future research questions derived from service user experience. Two people facilitated each group or individual meeting to enable effective note taking. The notes were shared at the end of each meeting and participants were then provided with contact information, and further invited to be involved in more formal PPIE panels if they wished.

### Collating findings and presenting stakeholder involvement

Following the meetings all notes were collated and discussed between researchers to determine the main findings about discharge and to draw conclusions from the suggestions and experiences, particularly in relation to the priorities that suggested further research focus. Further reading and analysis was undertaken to fully understand and to appreciate any differences and similarities between the views of individuals and groups, in addition to formulating priority issues as identified by the participants. The NIHR PPI report was formulated and presented to the NIHR in April 2015 further NIHR proposals have been submitted; based on the understanding of frail older adult priorities reported in this study.

### Findings

In total 27 individual responses were collected. The experiences of frail older NHS patients on admission and discharge are summarised as a series of topics to inform the continuing service improvements and to ensure that research planning was prioritised around patient experience. They are as follows:

### Priority 1: Reducing hospital length of stay

All participants appreciated the need for hospital stays to be as short as possible and if they were well enough they would prefer to be at home, even when their circumstances were not ideal. They agreed that continuing assessment at home was preferable to longer stays. Staying in hospital was also considered a problem because of the costly travelling and parking if patients were far from home.

*“Didn’t want to stay in hospital. Would be better off at home”*

*“Loneliness is dreadful. Still preferred to come home”*

Participants considered discharge planning to be highly individualised. People wanted concrete assurances to ensure that care and equipment would be in place when they arrived home.

*“I prefer home care to be in place before you come home - that would be good”*

*“How do I know that services will be there when I get home?”*

### Priority 2: Staying in hospital when necessary

As in other studies, patients sometimes felt they were pressurised to leave hospital too soon [23, 24] and wished to stay in hospital for longer until they felt better [25].

*“Sometimes you need to be in hospital”*

*“If you have no visitors at home or family, then it is better to be in hospital for a bit longer”*

The issue of assurance and some difficult experiences of the service were expressed, suggesting that the quality of care had caused some anxiety or they felt ‘rushed’ [23], whilst acknowledging that staff were under pressure to use their bed for another patient [25].

*“They were going to discharge me at 10pm, but I refused, and started to bleed later in the night”*

*“I was drugged and was not clear about what was happening and the Doctor didn’t tell me anything and I was discharged although I was feeling pain”*

*“Once you are ready to go, you want to go because when they are busy you can feel like you are in the way”*

### Priority 3: Staying independent

Many participants suggested that an important element of further assessment should include functional activity. A majority of participants expressed the desire for future research to prioritise projects investigating ways for the older adults to maintain their independence with daily living at home for as long as possible. Most could see the benefits of being assessed at home and motivated by the idea of personal independence:

*“If you don’t have home care, assessment at home is a good idea. I got good exercises from them. I liked the idea. They got me back walking”*

*“Whatever keeps your independence is a good thing. You lose your modesty in hospital.”*

“A*ssessment at home was more meaningful than in hospital”.*

### Priority 4: Appropriateness of D2A

There were some specific comments about the discharge process and these ideas were mixed and strongly related to individual social circumstances and personal feelings about what the hospital admission could achieve. There was apparently a shared view that those who were alone would need longer admissions.

*“If you live on your own and do not have support you might not like Discharge to Assess”*

*“Discharge to Assess was good for some but not others”*

*“Do a home visit to assess the person, and if they can’t manage then keep them a bit longer”*

### Priority 5: Communication processes

All patients expected clear communication between hospital staff and patients/families,. They especially wanted an opportunity to plan with relatives and carers for returning home.

*“Patients and families are not aware of the discharge plan at all”*

*“Tell the patients and their families about the discharge plan early not just before discharging”*

*“Sign post who to go to in the community for further advice”*

Participants stated that hospital staff sometimes made assumptions about the care that family members provide at home, especially in the BAME community. This could mean that questions related to home care are not asked by discharge planners.

*“Nobody asked me if there was help at home”*

*“Check who is at home and that they are well enough to look after you”*

### Priority 6: Maintaining research priorities

A more permanent PPIE panel was proposed at the end of interviews/ group sessions; suggesting that this could enable the voice of the older population to shape future research projects. It was thought to be a positive step forward, although none of the participants from this project ultimately became involved when a panel was established.

## Discussion

In summary, the frail older patients from across the city presented a range of views on hospital discharge. They assumed that staff would communicate with them in relation to discharge and make sure that social issues would be taken into account, but this was not always the case. The transitions between hospital and home encountered on discharge may represent many challenges but returning ‘home’ clearly has strong associations with being independent.

Engagement with patients and carers enabled a range of views to be gathered that could inform future research and service development. Results from the D2A project captured important perspectives from frail service users. A key finding was the wish to remain as independent as possible, which aligns to the top research priority identified by people with dementia from the James Lind Alliance [26]. As suggested by previous research findings that explore older adult views [27–29], our findings supported the belief that most people wish to be discharged home as soon as possible (priority 1), but this was qualified by the desire for the quality of the discharge to be equally as important and tailoring to individual needs is vital [30].

.There were some differences expressed within the participant groups in regard to communication, with members from the BAME community stating that assumptions were made in regard to their home support leading to a potential lack of formal services, something less likely to be stated by non BAME participants. Communication problems in general were expressed by many participants [31] regardless of ethnicity, and reflect similar problems raised by the adoption of D2A within a London Healthcare Trust, although these improved over time [32].

Other findings from the project revealed few differences in the responses of the participants regardless of socio-economic status, ethnicity or gender. The findings confirm some of the concerns raised by patients, including the frail older adults, in other studies. These included the need to take more notice of patient’s own perception of when they are ready for discharge, the need for increased formal support on discharge especially for the “older old” and those facing deteriorating life-limiting conditions, and improved partnership working between patients, their family and carers, and health and social care professionals. This applied both pre and post discharge [33–36] and views appear to have changed little over time.

In light of the findings, the challenge for those involved in discharge planning is to take greater account of patient’s own concerns, particularly in regard to their perceptions of when they are ready for discharge. The findings have been reported to the clinicians involved in D2A within the Trust enabling positive changes to be made to some of the processes involved with hospital discharge of the frail older people. The D2A participants suggest that future research needs to take account of the effect of earlier discharge on patients to result in a better patient experience [37], the latter being an NHS performance indicator which requires improvement [38]. This is particularly important to ensure the drive for service efficiencies and competing demands for resource allocation within healthcare do not adversely affect patient care and experience [4].

Earlier communication with family and carers may reduce incorrect assumptions made regarding the level of support at home, as well as giving family members reassurance about when their relative is to be discharged and with what level of professional support, allowing them to make necessary preparations themselves. An ongoing challenge is the increased demand for formal support, especially home care, with the increasing proportion of frail older population, at a time of financial restraint in social care budgets [39]. Without an appropriate increase in support, patients experiencing early discharge are likely to be readmitted [34, 40].

### Learning from the PPIE process

Participants for this project mainly attended focus groups despite the literature suggesting most older people prefer to be consulted individually in their own homes [41]. This was considered appropriate where participants lived within the same retirement complex and were therefore able to attend in familiar surroundings without the need to travel. Similarly, members of the PMC and over 50s club were happy to be consulted at their usual venues in a group situation, as they were familiar with their surroundings and each other, and could access them easily. Where people were not able to travel, or preferred not to participate in a group, individual interviews were offered. This was considered essential if a range of older adult voices were to be heard, and was a key consideration when planning future consultations with the participant group.

The PPIE project reinforced the understanding that frail older adults are not a homogenous group and a variety of methods to engage were offered to account for this. The challenge to undertake a sophisticated method of PPIE, requires a senior commitment to funded and co-ordinated activity as a part of research structures within a Trust. This level of commitment to PPIE is not universal. In addition the benefit of having a well-trained and supported PPIE group was evident in the successful recruitment to the D2A project. The advice from the initial PPIE group enabled a wider range of patients with experience of the D2A project to be included, ensuring views that reflected diverse communities. As a direct result of the PPIE project and the D2A public engagement the NHS Trust acknowledged that more permanent public involvement was required; prompting the formation of a sustainable older adult PPIE group, the Community Elders Panel (CEP). Aligned to the guidance provided by INVOLVE (the national advisory group supporting greater public involvement in the NHS) to identify, prioritise, design, conduct and disseminate NHS research [10], the CEP has been developed to support patient engagement and involvement in research, conducted within the Hospital Trust and local region.

The formation of the CEP was enabled by the NHS Trust committing to fund a part time PPIE coordinator. This provided the time and resources to recruit and train potential members and provide ongoing facilitation of the panel. Having succeeded in recruiting a range of participants for the D2A engagement following the advice of the original PPI group, similar methods were followed in recruiting to the CEP. Established community links and networks, as well as clinicians with links to older adult patients in the community were utilised to ensure a diverse range of panel members were recruited. It was hoped some of those who participated in the D2A engagement would join the CEP but this did not occur. The time frame from the initial project to the start of CEP recruitment was over 3 years which may account for this. Another factor may have been the D2A participants’ unfamiliarity with the PPIE coordinator who had not been involved in the original project. However, some of the contacts and venues used to recruit to the D2A project were used to successfully recruit to the panel.

The panel consists of patients and members of the public who are over 75, with some mobility issues and a wide range of health conditions and care needs. The panel is ethnically diverse; both genders are represented as are a wide range of socio-economic backgrounds. Sustainability has been achieved by providing bespoke training packages and consultations taking place in the members own homes, negating the need to travel and ensuring all individuals have the opportunity to be listened to. Visiting volunteer participants individually in their home environment has been shown to be successful in reaching very old and frail people [3].

### Impact

Ongoing support from the PPIE coordinator has enabled meaningful engagement with frail older housebound people, ensuring better representation of their views and priorities. Improved design of studies, more realistic outcome measures and research priorities with more relevance to older adults are some of the benefits gained so far from consulting the CEP. One of the research priorities identified by the D2A engagement was investigating methods to enable older adults to remain independent for longer, and the CEP have consulted on several studies involving these themes. The impact on panel members has also been positive; a feeling of contributing again to society, becoming more informed about the work and research being conducted within the Trust and having the opportunity to shape the direction of future healthcare were all benefits expressed by those taking part.

### Limitations

The findings from the D2A public engagement may not be generalizable to other areas. Efforts were made to ensure diversity when recruiting to both the D2A engagement and the CEP, however using gatekeepers may have inadvertently excluded some groups. The PPIE and follow-on activity benefitted from an institutional commitment to the formation and maintaince of formal panel activity and co-ordination within a care group. Results of the ongoing panel will be evaluated [42, 43].

## Conclusion

Three examples of PPIE with frail older adults have been described, each influencing and inspiring the next. The initial consultation with an existing PPIE group allowed more successful recruitment to the discharge planning (D2A) engagement project, both in terms of numbers and the diversity of those consulted. This engagement had two important outcomes. The first was to deepen the researchers understanding about critical issues for this patient population and the importance of safe and timely discharge from hospital. This is clearly a shared priority for services and for patients and carers. Patients recognised the need to plan their discharge in relation to family, social and community influences and saw the discharge process as an outcome of hospital treatment in relation to their own independence. The second was the perceived importance of learning directly from older people and ensuring their voices are heard. Establishing a sustainable PPIE infrastructure has enabled the views of older adults to be incorporated into the design, delivery and promotion of research projects and service redesign. Careful attention to gaining a range of views, especially from those seldom heard, has the potential to contribute to future success in the growing research programme in elderly medicine both nationally and internationally.

## Appendix

### Information sheet and topic questions. Patient and Public Involvement Process to investigate the views and experiences of patients and relatives being discharged from hospital.

□We are preparing to undertake some research and would like further information about the patient experience of discharge to inform our research question and design.

□We are aware that the “Discharge to Assess” practice aims to reduce the length of stay in hospital and that frail elderly people and their relatives may have a view about the length of stay and admission to hospital.

□We are also interested in how discharge is experienced by patients and carers of older people and what elements were helpful and what could be improved.

□Please complete the consent documentation saying that you understand that you are free to withdraw at any point and understand that we will be taking notes. Please could you provide feedback on the following;

□What was the experience of staying in hospital: awaiting discharge for some time or leaving hospital quickly?

□What do you think the NHS could learn from your experience?s.

□Do you have a preference in how you would like to be discharged if you were admitted again?

□Do you see longer or shorter hospital admissions as beneficial, and why?. We have a patient panel at the hospital, and would like to know if you would like to continue to help us develop our research questions and comment on proposals.

## Abbreviations

BAME: Black, Asian and Minority Ethnic
D2A: Discharge to assess
NHS: National Health Service
PMC: Pakistani Muslim Centre
PPIE: Public Involvement and Engagement
RDS Y&H: Regional Design Service of the NIHR in Yorkshire and Humber
RfPB: Research for Patient Benefit
